# Supplementary material for: Detection and functional resolution of soluble immune complexes by an FcγR reporter cell panel
Source: EMBO Mol Med. 2021 Nov 29;14(1):e14182. doi: 10.15252/emmm.202114182 (PMC8749491; doi:10.15252/emmm.202114182)
Supplement: Supplementary file 1 — Expanded View Figures PDF [file EMMM-14-e14182-s001.pdf]

Expanded View Figures

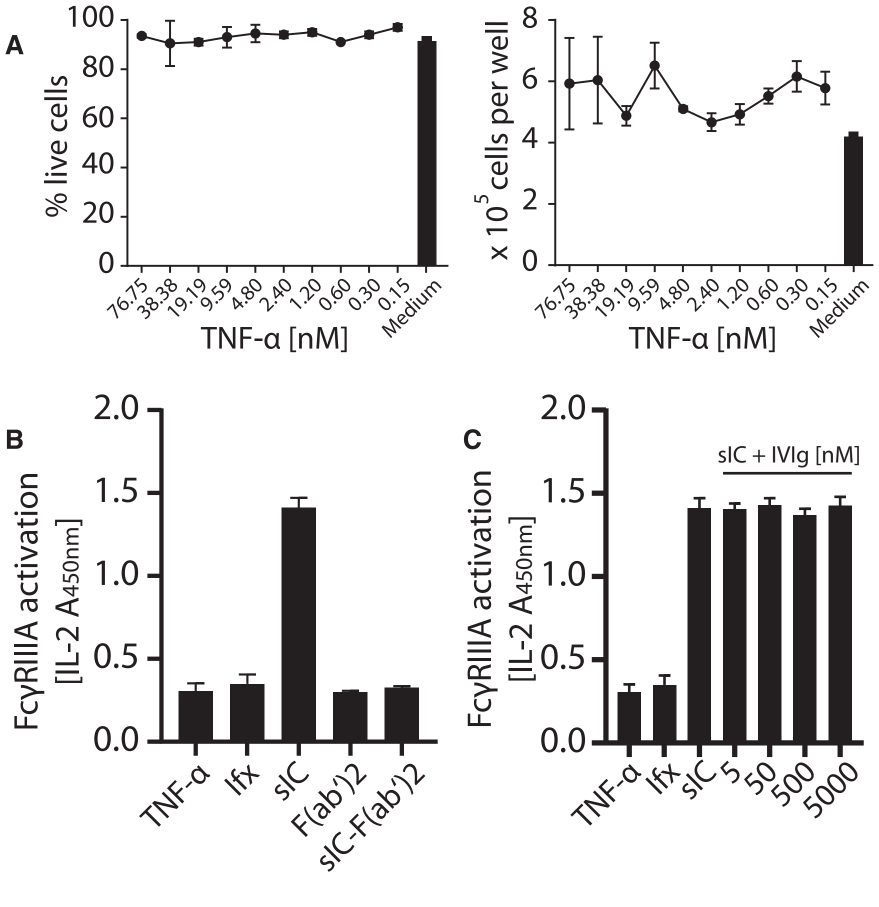

Figure EV1. Controls for reporter cell specificity and tolerance to IVIg.

A Human TNF-α is not toxic to mouse lymphocyte BW5147 cells even at high concentrations. Cell count and percentage of live cells were unaltered over a 16 h time frame of reporter cell culture in the presence of indicated TNF-α concentrations and comparable to regular growth in complete medium. One experiment performed in three technical replicates. Error bars = SD.

B Fcγ is required for reporter cell activation; 100 nM TNF-α was mixed with 50 nM infliximab or F(ab′)2 fragments of infliximab (Ifx) to generate sICs. TNF-α, Ifx or Ifx-F(ab′)2 fragments alone served as negative controls. One experiment performed in four technical replicates. Error bars = SD.

C Reporter cell activation by sIC is tolerant to high amounts of non-complexed monomeric IgG. Reporter cell activation by TNF-α:Ifx sICs as in (B) was challenged by the addition of titrated amounts of IVIg. TNF-α or Ifx alone served as negative controls. One experiment performed in four technical replicates. Error bars = SD.

 © 2021 The Authors

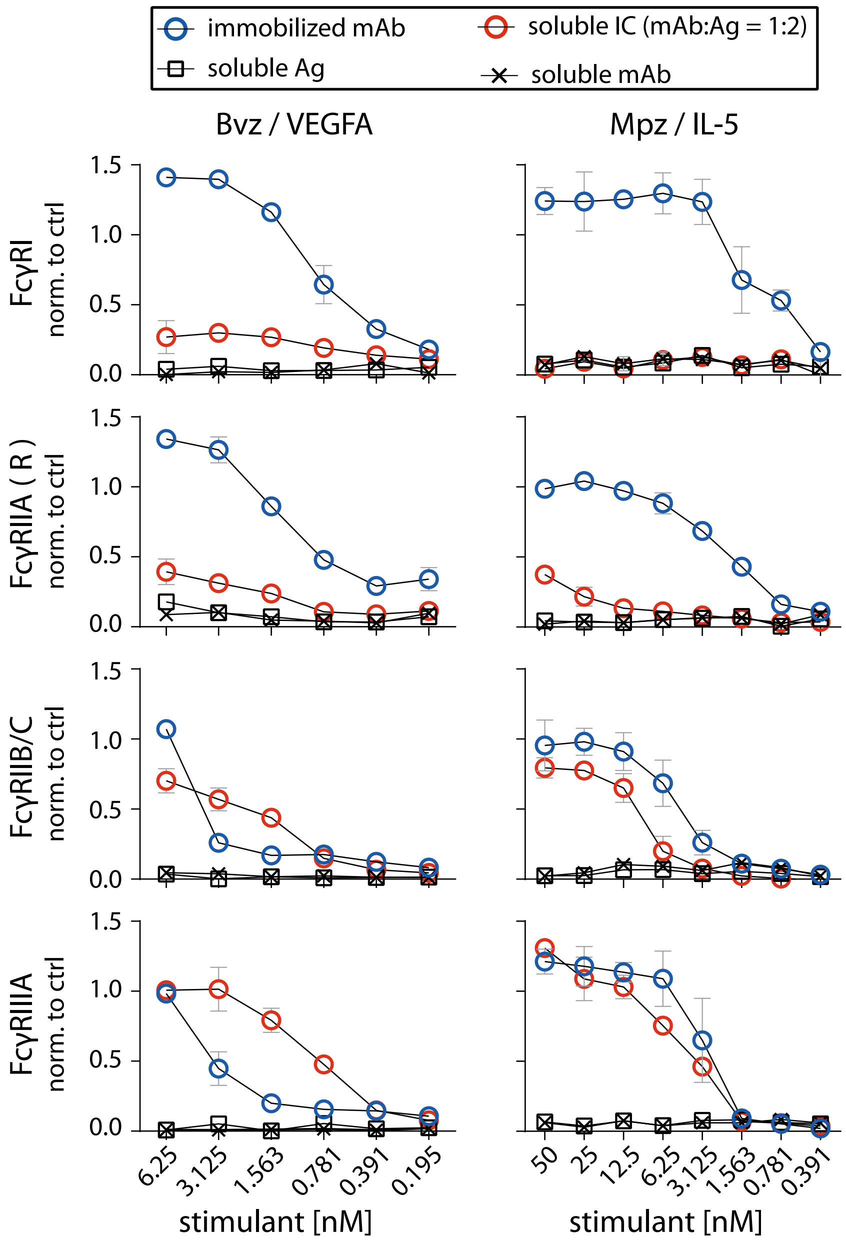

Figure EV2. FcγRs are activated by sICs formed from multivalent antigens.

Two different multivalent ultra-pure antigens (Ag) mixed with respective therapy-grade mAbs were used to generate sICs as indicated for each set of graphs (top to bottom). IC pairs: mepolizumab (Mpz) and rhIL-5; bevacizumab (Bvz) and rhVEGFA. X-Axis: concentrations of stimulant expressed as molarity of either mAb or Ag monomer and IC (expressed as mAb molarity) at a mAb:Ag ratio of 1:2. Soluble antigen or soluble antibody alone served as negative controls and were not sufficient to activate human FcγRs. FcγR responses were normalized to immobilized rituximab (Rtx) at 1 µg/well (set to 1) and a medium control (set to 0). Two independent experiments performed in technical duplicates. Error bars = SD. Error bars smaller than symbols are not shown.

© 2021 The Authors

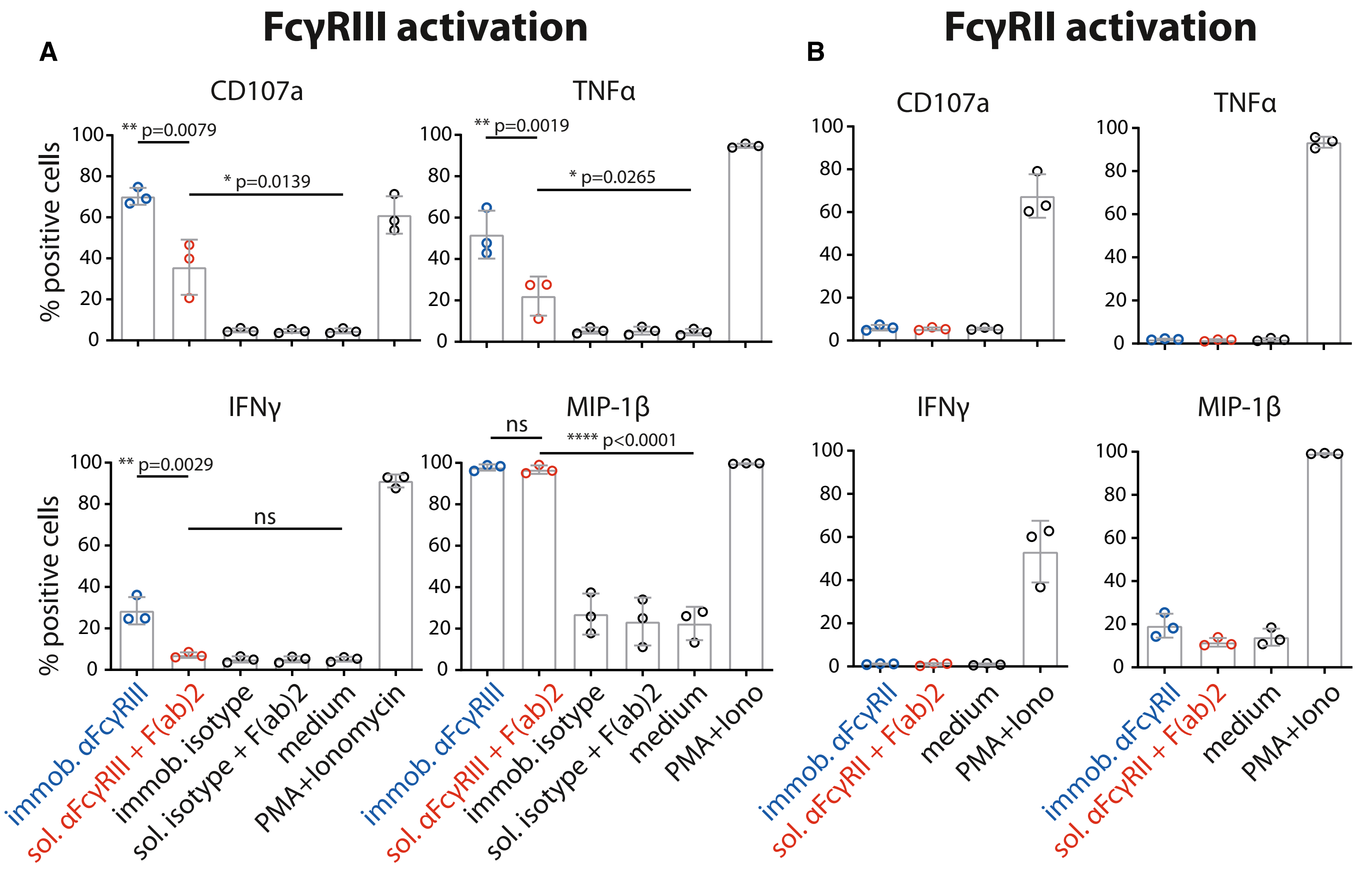

Figure EV3. Distinct activation patterns of NK cells incubated with inverse sICs.

Negatively selected primary NK cells purified from PBMCs of three healthy donors were tested for NK cell activation markers.

A NK cells were incubated for 4 h with immobilized FcγRIII-specific mAb, soluble mouse-anti-human IgG F(ab′)2 complexed FcγRIII-specific mAb (reverse sICs), immobilized IgG of non-FcγRIII-specificity (isotype control) or soluble F(ab′)2 complexed isotype control (all at 1 μg, 10^6 cells). Incubation with PMA and Ionomycin served as a positive control. Incubation with medium alone served as a negative control.

B As in A using an FcγRII-specific mAb. NK cells from the tested donors in this study do not react to FcγRII activation.

Data information: Each dot represents one donor measured in technical duplicates. Error bars = SD. One-way ANOVA (Tukey); *$P < 0.05$, **$P < 0.01$, ****$P < 0.0001$.

© 2021 The Authors
