# Supplementary material for: Detection and functional resolution of soluble immune complexes by an FcγR reporter cell panel
Source: EMBO Mol Med. 2021 Nov 29;14(1):e14182. doi: 10.15252/emmm.202114182 (PMC8749491; doi:10.15252/emmm.202114182)
Supplement: Supplementary file 2 [file EMMM-14-e14182-s002.pdf]

EMBO Molecular Medicine

Detection and functional resolution of soluble immune complexes by an FcγR reporter cell panel

Haizhang Chen, Andrea Maul-Pavicic, Martin Holzer, Magdalena Huber, Ulrich Salzer, Nina Chevalier, Reinhard Voll, Hartmut Hengel, and Philipp Kolb

DOI: 10.15252/emmm.202114182

Corresponding authors: Philipp Kolb (philipp.kolb@uniklinik-freiburg.de) , Hartmut Hengel (Hartmut.hengel@uniklinik-freiburg.de)

Review Timeline:

Submission Date:	25th Feb 21
Editorial Decision:	4th Mar 21
Author Correspondence:	9th Mar 21
Editorial Correspondence:	11th Mar 21
Revision Received:	18th Mar 21
Editorial Decision:	29th Apr 21
Author Correspondence:	1st Sep 21
Editorial Correspondence:	2 Sep 21
Revision Received:	15th Sep 21
Editorial Decision:	4th Oct 21
Revision Received:	4th Nov 21
Editorial Decision:	5th Nov 21
Revision Received:	8th Nov 21
Accepted:	9th Nov 21

Editor: Zeljko Durdevic

Transaction Report:

(Note: With the exception of the correction of typographical or spelling errors that could be a source of ambiguity, letters and reports are not edited. Depending on transfer agreements, referee reports obtained elsewhere may or may not be included in this compilation. Referee reports are anonymous unless the Referee chooses to sign their reports.)

25th Feb 2021

Decision on your manuscript EMM-2021-14182

Dear Dr. Hengel,

Thank you for submitting your manuscript to EMBO Molecular Medicine. Upon receipt, manuscripts can sometimes be evaluated by the Scientific Editors to deal in a timely fashion with a large number of submissions. In this case, I am afraid that we concluded that your manuscript is not well suited for publication in EMBO Molecular Medicine and have therefore decided not to proceed with peer review.

While potentially of interest to the more immediate community, I am afraid that due to its nature, the article doesn't fit well within EMBO Molecular Medicine as we focus primarily on these studies that provide functional novel insights of clinical and/or translational significance, but also that are conceptually novel and of broad interest. As we do not feel that this is the case here, we therefore cannot offer further consideration to your manuscript.

I am sorry to have to disappoint you on this occasion; in the interest of time, I am providing you with an early decision that will allow you to submit your manuscript elsewhere without any further delays.

Please rest assured that this is not a judgment of the quality or interest of your work but a decision based on appropriateness for EMBO Molecular Medicine.

Yours sincerely,

Zeljko Durdevic

Zeljko Durdevic
Editor
EMBO Molecular Medicine

Dear Dr. Durdevic,

on the 29th of April we received reviews for our manuscript (EMM-2021-14182-V3). Although the submission was met with a negative decision, we appreciate the in-depth reviews that inspired us to put great effort into the adaptation and thorough revision of our manuscript. We were able to fully address the concerns about our work and are able to now provide a substantially improved manuscript. As we are still convinced that EMBO: Molecular Medicine is the most fitting journal for the publication our work, we would be very happy if you were to support a resubmission.
We are confident, that the manuscript is of great interest to the readership of EMBO: Molecular Medicine and that this improved study now meets the requirements for publication as a Resource/Method article in your journal.

Major changes to the original submission are listed below:

1) The manuscript was revised to be more focused and clear, which is reflected in a new title and a reduction in the number of figures in the main text

2) We expanded our reporter cell line-up to now feature all human Fc-gamma receptors and collected more data on the validation of the assay using primary cells (NK cells and now also Neutrophils)

3) We now provide direct assay quantification using a titrated standard regarding the original readout (Il-2 via ELISA - 16 h incubation)

4) We now also provide an alternative read-out for faster measurements (CD69 via flow cytometry - 4 h incubation)

Best regards,
Philipp Kolb and Hartmut Hengel

11th Mar 2021

Dear Dr. Hengel,

Thank you for your response to the editorial decision on your manuscript entitled "Immune complex solubility and size govern Fc-gamma receptor responses". We have carefully examined the arguments provided in your letter and feel that we could consider your manuscript as a Resource/Method article for peer review in EMBO Molecular Medicine. If you agree, please submit the adapted version of the manuscript mentioned in your appeal letter and indicate the changes. I look forward to receiving your manuscript.

Yours sincerely,

Zeljko Durdevic

Zeljko Durdevic
Editor
EMBO Molecular Medicine

Dear Dr. Durdevic,

thank you very much again for your favourable response to our appeal request.

We have submitted a slightly adapted manuscript via the link provided in your email. As we did not yet enter in-depth review with our manuscript, we treated the upload similar to an initial submission, providing single files with integrated figures. We hope this is in line with your expectation at this stage of the submission. If not, please let us know, we will respond to your standards immediately.

The changes made to the manuscript are detailed in an added paragraph to our cover letter, which we again provide with all its original content.

Sincerely yours,

Hartmut Hengel and Philipp Kolb

Professor Hartmut Hengel, MD
Head, Institute of Virology
Department of Medical Microbiology and Hygiene
University Medical Center
Albert-Ludwigs-University Freiburg
Hermann-Herder-Strasse 11
D-79104 Freiburg
Germany
Phone: 49 761 203 6533 or 6534
FAX. 49 761 203 6626
hartmut.hengel@uniklinik-freiburg.de<mailto:hartmut.hengel@uniklinik-freiburg.de>

29th Apr 2021

Decision on your manuscript EMM-2021-14182-V3

Dear Dr. Kolb,

Thank you for the submission of your manuscript to EMBO Molecular Medicine. We have now received feedback from the three reviewers who agreed to evaluate your manuscript.

As you will see from their reports pasted below, while they recognize interest of your study, they also raise serious and overlapping critique that preclude further consideration of the article. As clear and conclusive insight into a novel, clinically relevant observation is crucial for publication in EMBO Molecular Medicine, I am afraid that we cannot offer to consider the manuscript further.

I am sorry that I could not bring better news this time and hope that the referee comments are helpful in your continued work in this area.

Yours sincerely,

Zeljko Durdevic

Zeljko Durdevic
Editor
EMBO Molecular Medicine

***** Reviewer's comments *****

Referee #1 (Comments on Novelty/Model System for Author):

The system used by the authors is not novel. Already detailed bin other reports and authors adapted the system for their work. There are well characterized biomarkers for autoimmune diseases like lupus and usefulness of the methodology for clinical investigation is not clear.

Referee #1 (Remarks for Author):

The system used by the authors is not novel. Already detailed in other reports and authors adapted the system for their work. There are well characterized biomarkers for autoimmune diseases like lupus and usefulness of the methodology for clinical investigation is not clear.

The signaling pathways mediated by sIC upon interaction with various FcgammaR is important. Also the role of inhibitory ITAM

The expression of Fcgamma receptors in the patients varies depending on the cytokines, inflammation and genetic background. Therefore, net results obtained with reporter vs natural conditions might not be the same

Glycosylation of IgG in the IC is important to consider in the experiments.

The role of variant forms of Fcgamma receptors need to be explored

Referee #2 (Comments on Novelty/Model System for Author):

The experiments performed by Chen and colleagues appear to have been well-conducted. However, in many cases, it is unclear from the figure legends how many times the indicated experiments were performed. An example of this is in the legend for Figure 2, where what the error bars represent is also unclear. When it is stated that "experiments performed in technical

replicates", for example in the legend for Figure 3, is this from a single experiment? In other cases, the details of statistical analysis are unclear. For example, in Figure 4, what levels of significance do the **s correspond to? All legends should clearly demarcate the number of biological (experimental) replicates to provide confidence of reproducibility and the variability between experiments. Similarly, it should be explicit what statistical tests are being performed, what bars and errors are and which groups are being compared

The reporter cell system involved has previously been published by the authors but here the authors modify it to ensure that only soluble immune complexes are being detected (largely by pre-incubation with FCS).

The detection of immune complexes itself is not novel and alternative assays have been developed over the years such as that by Stopforth et al (2018). However, unlike the former paper, here the authors use activatory Fc gamma receptor IIIa-dependent responses coupled to IL-2 secretion as a read out, unlike the inhibitory Fc gamma receptor IIb signalling used by Stopforth and colleagues. Moreover, Chen and colleagues have also explored some additional aspects, such as immune complex size. They have also performed experiments with primary immune cells in an attempt to validate the reporter assay results (for Fc gamma receptor IIIa), and expanded the methodology to detect mouse IgG immune complex binding to mouse Fc gamma receptors.

Chen and colleagues discuss how the assay could be used to detect pathological immune complexes within serum samples of autoimmune disease patients, thereby meeting an unmet clinical need. However, whether this assay could be easily integrated into routine clinical practice/ diagnostics is unclear. For example, 16 hours of incubation of immune complexes with reporter cells would be required, followed by an ELISA to detect IL-2. How do the authors propose that the assay could be used within a clinical setting, considering these limitations? It would be helpful if the authors could expand on the current state of the art (how are IC measured clinically currently?), other alternative assays and how their system provides an advantage. Also, how do the authors address the issue reported on page 6 "only combinations of pharmaceutical therapy grade, i.e. ultra-pure mAbs and pure antigens (purified via size exclusion chromatography) showed reproducible, dose-dependent and specific activation in the reporter assay (data not shown)."

Similarly, the reporter output is OD450nm. Presumably this requires conversion to IL-2 concentration and with reference to a specific standard curve (taking in to account the difference in response shape with different immune complexes sizes and components (even purified monoclonals) - it would help if the authors could expand on what steps are required to make the assay suitable for clinical use.

Chen and colleagues have expressed chimeric human Fc gamma receptors in reporter cells. They have also performed work with primary NK cells to show Fc gamma receptor IIIa-dependent responses and thereby recapitulate the observations from their reporter assays.

I have a small issue in understanding the key principle of the reporter cells. According to Figure 1A they display a 2 log difference in their ability to bind Fc (IIB/C much lower than IIIA). Similarly, whereas FcgRI and IIa/B are stained equivalently with FITC, the FcgRIII reporters are stained with PE. How are we supposed to interpret this and then consider differences between FcgR signalling if they differ in expression level? (NB: I am also presuming that the atypical 1,2,3,4 show on the x axis for figure 1 is representing a log scale of fluorescence?). The FcgRI cells have a very broad expression curve (reaching to the negative control whereas the FcgRIIB are much tighter and higher. What is the geometric mean of their expression levels? In summary, how do the authors control for differences in expression level? Could they assess numbers of receptors on each cell-line for example?

Like the authors, I am surprised by the absence of reporter cell responses with Fc gamma receptor I- and IIa-expressing reporter cells and consider this a key finding. The authors have explained this observation by referencing several articles, but I still fail to understand the rationale: IIA is a transmembrane receptor containing an ITAM - very similar to the chimera and contains a 93-97% identical extracellular domain to IIB, which does signal in the assay. FcgRI - requires co-associated gamma chain for signalling, akin to IIIA and again doesn't signal whereas IIIA does. In fact, Promega already sell an FCGR2A-mediated "adcp" reporter assay based on luciferase read-out as well as an FCGR3A reporter. Could the authors therefore perform a similar approach and test conclusively that the FCGR1 and 2A lack of detection is not an unexpected oddity of the IL-2 reporter/BW5147 cells. i.e. using the same assay system with FCS blockade and then the promega reporter cells?

Bruhns et al. 2009 clearly showed that Fc gamma receptor I and IIa bind to immune complexes. In the work of Stopforth et al. (2018), the results indicated that Fc gamma receptor IIa signalling was required for activation of the inhibitory Fc gamma receptor IIb. Other literature has also suggested that immune complex binding to Fc gamma receptor IIa is functional. Therefore, are soluble immune complexes not detected in the assays described because of an absence of binding to Fc gamma receptor I and IIa, or some other reason such as the nature of the Fc gamma receptor chimeras or immune complexes being tested? More importantly, if the soluble immune complexes bind to FcgRI and IIa but do not signal, does this mean that there could be a false negative readout when detecting IgG immune complexes in autoimmune disease sera with Fc gamma receptor I or IIa expressing reporter cells? To this end, do the authors propose that Fc gamma receptor IIIa-expressing reporter cells would be the reporter cell of choice for the detection of pathologic immune complexes?

The authors also state that "As this assay is highly sensitive to any aggregation of IgG, it also represents a tool to control the

purity, quality and stability of mAb preparations produced fortherapeutic use in patients." - apologies but I could not find data to support this claim.

The article is well-written and clear, and key concepts are well explained (notwithstanding some technical facts detailed below). It is my opinion that a non-specialist would both be able to understand the article and find it interesting.
However a couple of sentences would benefit for greater explanation - e.g. "functional reproduction of the paradigmatic Heidelberger- Kendall precipitation curve, describing that the molecular size of sICs determined by the antibody:antigen ratio". This concept is described in the abstract also but is not adequately explained.

Similarly this concept is not explained or introduced "Among all type I FcγRs,"

There are several statements in the introduction that are either incomplete and/or to my mind incorrect.

For example - no mention of the binding of complement C1q is given in the following sentence - "and the constant Fc region (Fcγ), which is detected by Fcγ receptors (FcγRs) found on 43 most cells of the immune system". This should be rectified for balance.

"When IgG binds to its antigen ICs are formed, which, depending on the respective antigen, are either cell-bound or soluble (sICs)." - IgG can also bind structural and matrix proteins that are not inherenly cellular or soluble

This statement is unclear" Firmly fixed cell bound ICs" - what does that mean?

This statement is controversial "FcgR1....is primarily tasked with phagocytosis linked to antigen processing and pathogen clearance" and should be removed.

Similarly the statements about the definitive roles of each FcgR are not accurate or well accepted and should be amended "Activation of FcγRs leads to a variety of cellular effector functions such as antibody-dependent cellular cytotoxicity (ADCC) by natural killer (NK) cells via FcγRIIIA, antibody-dependent cellular phagocytosis (ADCP) by macrophages via FcγRI, cytokine and chemokine secretion by NK cells and macrophages via FcγRIIIA." - many of these statements are isotype, assay and context dependent.

As indicated above, some of the themes of the paper have been covered in previously published literature (Stopforth et al. 2018). For this reason, care should maybe be taken with the sentence "...that is able for the first time to selectively detect soluble multimeric immune complexes as discrete ligands of FcγR". However, certainly some novel aspects have been explored in the paper, including the recapitulation of the Heidelberger Kendall precipitation curve, work with primary NK cells, and the detection of mouse IgG immune complexes. Finally, considering the time required to perform such assays, it would be important to clearly define how the assay would be able to be used in a clinical setting.

Some referencing is lacking - where did the "monomeric CD20 peptide (aa 141-188)" knowledge come from?

Referee #3 (Remarks for Author):

The authors developed a reporter cell-based system that evaluates FcgR activation by sICs as a tool to quantitate sIC activity. They show that sIC generated in vitro, using synthetic dimeric or trimeric antigens, and present in sera of lupus mice and patients are detected by cell lines expressing the ectodomain of the human FcgR fused to the signaling module of the mouse CD3-zeta chain. They also exploited previously described FcgR reporter cells expressing chimeric mouse FcgRs. The readout of activation is the generation of IL-2 detected in the cell culture. Thus, sIC mediated activation rather than binding to FcgRs per se is being measured. The experiments are technically well done and shows sensitivity to sIC stoichiometry and size (antibody-antigen ratio). However, the objective for this study is not clear. Is it to identify the FcgRs that do not respond to soluble ICs or to develop a reporter system to detect sICs in clinical samples? Both have caveats as described below.

Using the reporter cell panel expressing individual human and mouse FcgRs, the authors claim that sICs do not activate FcgRI or FcgRIIA. The latter is quite surprising and the authors don't explain how FcgRIIA is not, while FcgRIIB/C is activated by sICs when both FcgRs are highly homologous in their extracellular domain. In this regard, the statement that FcgRIIA is unable to recognize sICs (line 73) is incorrect (i.e. Fossati et al. 2002 and Hubbard et al. 2020 are not accurately quoted).

The mouse homolog for human FcgRIIA is mFcgRIII and for human FcgRIII is mFcgRIV. However, despite a lack of activation of FcgRIIA by sICs, mFcgRIV responsiveness tended to be lower and more variable than FcgRIII.

The use of human IgG-antigen pairs that provide dimeric or trimeric antigen are innovative but it is not clear that this approach is superior to the NIP-antigen system used by Lux et al 2016. It is also not clear how the current studies advances the field as the

principle that the molecular size of sICs dynamically tunes FcgR activation (line 59) was explored by Lux et al. 2016.

If the objective is to develop the reporter cell-based assay as a clinical tool then the authors need to demonstrate that it outperforms current clinical assays and that it is scalable and high-throughput for clinical labs. In addition, the development of a reporter cell line that expresses multiple FcgRs (as is the case for mammalian leukocytes) would be warranted.

Dear Dr. Durdevic,

on the 29th of April we received reviews for our manuscript (EMM-2021-14182-V3). Although the submission was met with a negative decision, we appreciate the in-depth reviews that inspired us to put great effort into the adaptation and thorough revision of our manuscript. We were able to fully address the concerns about our work and are able to now provide a substantially improved manuscript. As we are still convinced that EMBO: Molecular Medicine is the most fitting journal for the publication our work, we would be very happy if you were to support a resubmission.
We are confident, that the manuscript is of great interest to the readership of EMBO: Molecular Medicine and that this improved study now meets the requirements for publication as a Resource/Method article in your journal.

Major changes to the original submission are listed below:

1) The manuscript was revised to be more focused and clear, which is reflected in a new title and a reduction in the number of figures in the main text

2) We expanded our reporter cell line-up to now feature all human Fc-gamma receptors and collected more data on the validation of the assay using primary cells (NK cells and now also Neutrophils)

3) We now provide direct assay quantification using a titrated standard regarding the original readout (Il-2 via ELISA - 16 h incubation)

4) We now also provide an alternative read-out for faster measurements (CD69 via flow cytometry - 4 h incubation)

Best regards,
Philipp Kolb and Hartmut Hengel

Dear Dr. Kolb,

thank you for your e-mail. I am happy to hear that you were able to fully address the referees' concerns and thank you for highlighting the major changes in your revised manuscript. Your request to resubmit the revised version of your manuscript will be considered as an appeal to the editorial decision. However, in order to adequately respond to your inquiry we would need more information. Therefore, please send the revised manuscript and point-by-point response for editorial evaluation. Thank you very much.

Best wishes,
Zeljko

Zeljko Durdevic, PhD
Editor
EMBO Molecular Medicine
z.durdevic@embomolmed.org

Point by point reply to reviewers' comments:

Referee #1 (Remarks for Author):

The system used by the authors is not novel. Already detailed in other reports and authors adapted the system for their work. There are well characterized biomarkers for autoimmune diseases like lupus and usefulness of the methodology for clinical investigation is not clear.

Reply #1: We feel that the novelty of our system was not presented in the right context and now clearly explain how the system sets itself apart from previous assays, where its limitations are and where it can be applied (in the abstract, lines 102-106, lines 317-324 and from line 361). In general, compared to previous applications of the BW5147 FcγR reporter cells, the adapted assay allows for the first time the selective measurement of the bioactivity of soluble multimeric immune complexes (sICs) instead of immobilized immune complexes. In the revised manuscript, we now also provide a larger panel of BW5147 reporter cells including all human FcγRs and quantification of the reporter activation (new Fig. 2). Further, the new manuscript provides a previously not-described readout allowing for faster evaluation of sIC bioactivity in high-throughput flow cytometry (new Fig. 2). Compared to other biomarker-assays for lupus, our approach provides a more sensitive, scalable and fast addition measuring bioactivity of sICs. Bioactivity is a feature previously not detected with commercial assays. However, we agree, that the platform is not immediately applicable in a routine clinical setting. In the new manuscript, we toned down our speculation on application in clinical settings. We rather point out the advantages for possible application in clinical settings. Further, we now also highlight the usefulness of the approach in experimental and clinical research.

The signaling pathways mediated by sIC upon interaction with various FcgammaR is important. Also the role of inhibitory ITAM

Reply #2: We agree that using a reporter cell line of murine origin is a reductionistic approach for the sensitive detection of sICs but does not reflect the cell biology of primary human immune cells and cannot fully reflect all aspects of immune cell activation. We now clearly mention the limitations and advantages of using a reporter system (from line 361).

The expression of Fcgamma receptors in the patients varies depending on the cytokines, inflammation and genetic background. Therefore, net results obtained with reporter vs natural conditions might not be the same

Reply #3: We fully agree with the reviewer and now make clearer that the reporter system is unique and useful for detection, quantification and bioactivity-measurement

of sICs and their functional assignment to a defined FcγR type. To address this point further, we now also tested sIC-mediated neutrophil activation to compare our assay to primary cell responses (new Figure 3B). Together with the data obtained from primary NK cells, this more clearly shows to what extent the reporter assay reflects the activation of primary cells and where its limitations are.

Referee #2 (Comments on Novelty/Model System for Author):

The experiments performed by Chen and colleagues appear to have been well-conducted. However, in many cases, it is unclear from the figure legends how many times the indicated experiments were performed. An example of this is in the legend for Figure 2, where what the error bars represent is also unclear. When it is stated that "experiments performed in technical replicates", for example in the legend for Figure 3, is this from a single experiment? In other cases, the details of statistical analysis are unclear. For example, in Figure 4, what levels of significance do the **s correspond to? All legends should clearly demarcate the number of biological (experimental) replicates to provide confidence of reproducibility and the variability between experiments. Similarly, it should be explicit what statistical tests are being performed, what bars and errors are and which groups are being compared.

Reply #4: We thank the reviewer for his/her positive and appreciative assessment of our work, but also for pointing out specific flaws. The text of the legends to the mentioned figures was thoroughly revised and the required information is now clearly stated in the legends to all figures.

The reporter cell system involved has previously been published by the authors but here the authors modify it to ensure that only soluble immune complexes are being detected (largely by pre-incubation with FCS). The detection of immune complexes itself is not novel and alternative assays have been developed over the years such as that by Stopforth et al (2018). However, unlike the former paper, here the authors use activatory Fc gamma receptor IIIa-dependent responses coupled to IL-2 secretion as a read out, unlike the inhibitory Fc gamma receptor IIb signalling used by Stopforth and colleagues. Moreover, Chen and colleagues have also explored some additional aspects, such as immune complex size. They have also performed experiments with primary immune cells in an attempt to validate the reporter assay results (for Fc gamma receptor IIIa), and expanded the methodology to detect mouse IgG immune complex binding to mouse Fc gamma receptors.

Reply #5: We agree that the detection of sICs per se is not new. In order to set our comprehensive assay system more apart from previous approaches (see reply #1 to reviewer 1).

Chen and colleagues discuss how the assay could be used to detect pathological immune complexes within serum samples of autoimmune disease patients, thereby meeting an unmet clinical need. However, whether this assay could be easily integrated into routine clinical practice/ diagnostics is unclear. For example, 16 hours of incubation of immune complexes with reporter cells would be required, followed by an ELISA to detect IL-2. How do the authors propose that the assay could be used within a clinical setting, considering these limitations? It would be helpful if the authors could expand on the current state of the art (how are IC measured clinically currently?), other alternative assays and how their system provides an advantage.

Reply #6: We thank the reviewer for this inspiring criticism. We now provide additional readouts to the assay (new Fig. 2). Quantification of IL-2 secretion and a much faster (4h) high-throughput readout by measuring CD69 expression via flow cytometry. In addition, we now state the limitations and advantages of the assay more clearly (from line 361). We also now evaluate the assay in comparison with available C3d or C1q-CIC assays (in the abstract, lines 102-106, lines 317-324 and from line 361). In practice, hese assays are CE-certified but not routinely used. One practical reason is their limited scalability, which is an advantage of our system. Specifically, the faster FACS-based read-out can be scaled from single samples to a 96-well plate format.

Also, how do the authors address the issue reported on page 6 "only combinations of pharmaceutical therapy grade, i.e. ultra-pure mAbs and pure antigens (purified via size exclusion chromatography) showed reproducible, dose-dependent and specific activation in the reporter assay (data not shown)."

Reply #7: The reviewer is right; this is a "limitation" of the assay highlighting the exquisite sensitivity and specificity of the assay system, which does not tolerate the presence of artificially in vitro aggregated IgG. However, this issue regards the experimental set up of the assay, its validation in basic research and its application in quality assurance of pharmaceutical products such as immune sera and recombinant antibodies. In contrast to native clinical samples (e.g. serum samples from patients with pathogenic sICs such as SLE patients and COVID-19 patients, joint fluid from RA patients and freshly taken sera from healthy controls,) are not affected by precipitated IgG aggregates.

Similarly, the reporter output is OD450nm. Presumably this requires conversion to IL-2 concentration and with reference to a specific standard curve (taking in to account the difference in response shape with different immune complexes sizes and components (even purified monoclonals) - it would help if the authors could expand on what steps are required to make the assay suitable for clinical use.

Reply #8: We thank the reviewer for the constructive suggestion. IL-2 quantification

was added (new Fig. 2). We now also provide a faster readout using high-throughput flow cytometry, more suitable for clinical sIC detection (new Fig. 2). We also clearly state the limitations of the assay (from line 361).

Chen and colleagues have expressed chimeric human Fc gamma receptors in reporter cells. They have also performed work with primary NK cells to show Fc gamma receptor IIIa-dependent responses and thereby recapitulate the observations from their reporter assays. I have a small issue in understanding the key principle of the reporter cells. According to Figure 1A they display a 2 log difference in their ability to bind Fc (IIB/C much lower than IIIA). Similarly, whereas FcgRI and IIa/B are stained equivalently with FITC, the FcgRIII reporters are stained with PE. How are we supposed to interpret this and then consider differences between FcgR signalling if they differ in expression level? (NB: I am also presuming that the atypical 1,2,3,4 show on the x axis for figure 1 is representing a log scale of fluorescence?). The FcgRI cells have a very broad expression curve (reaching to the negative control whereas the FcgRIIB are much tighter and higher. What is the geometric mean of their expression levels? In summary, how do the authors control for differences in expression level? Could they assess numbers of receptors on each cell-line for example?

Reply #9: We agree with the reviewer that this was not made clear enough. In the new manuscript, we now provide more information on reporter cell generation (adapted Fig. 1). We also measured all reporter cells with the same dye and compare MFIs as well as receptor positivity. FcγR surface detection is not equal and receptor density varies to some extent between reporter cell lines. This might be due to using different antibodies for detection due to inherent features of the FcγRs (e.g. supported by differences between CD32 reporters measured using the same antibody). Therefore, we do not directly compare individual FcγR cell lines with one another but only compare their individual activation patterns. We considered introducing N-terminally tagged FcgRs into the reporter cell system to address this. However, this would require extensive evaluation regarding comparability with untagged receptors. Naturally, with a complete reporter panel in hand, this is now possible.

Like the authors, I am surprised by the absence of reporter cell responses with Fc gamma receptor I- and IIa-expressing reporter cells and consider this a key finding. The authors have explained this observation by referencing several articles, but I still fail to understand the rationale: IIA is a transmembrane receptor containing an ITAM - very similar to the chimera and contains a 93-97% identical extracellular domain to IIB, which does signal in the assay. FcgRI - requires co-associated gamma chain for signalling, akin to IIIA and again doesn't signal whereas IIIA does. In fact, Promega already sell an FCGR2A-mediated "adcp" reporter assay based on luciferase read-out as well as an FCGR3A reporter. Could the authors therefore perform a similar approach and test conclusively that the FCGR1 and 2A lack of detection is not an unexpected oddity

of the IL-2 reporter/BW5147 cells. i.e. using the same assay system with FCS blockade and then the promega reporter cells?

Reply #10: The reviewer made an excellent point which motivated us to broaden our reporter line-up (added FCGR3B and FCGR2A[H]), re-select and FACS-sort our reporter cells and measure again (new Fig. 2). This revealed that our previous observation regarding FCGR2A was exaggerated. While rough trends remained as before, using the new FCGR2A (R vs H) reporter cells allowed for a clearer interpretation of the data and gives a more complete picture. The FCGR1 cells, however, remained to be very low in responsiveness to sICs. We now discuss this observation differently as we were not able to reach comparably high receptor densities on the reporter cells (lines 168-172). As we were limited regarding the lupus serum material, we did not revisit this experiment with the new cells but now removed the data obtained with the FCGR1 and FCGR2A(R) cells. In general, the manuscript is now written more clearly as a resources/methods article.

Bruhns et al. 2009 clearly showed that Fc gamma receptor I and IIa bind to immune complexes. In the work of Stopforth et al. (2018), the results indicated that Fc gamma receptor IIa signalling was required for activation of the inhibitory Fc gamma receptor IIb. Other literature has also suggested that immune complex binding to Fc gamma receptor IIa is functional. Therefore, are soluble immune complexes not detected in the assays described because of an absence of binding to Fc gamma receptor I and IIa, or some other reason such as the nature of the Fc gamma receptor chimeras or immune complexes being tested? More importantly, if the soluble immune complexes bind to FcgRI and IIa but do not signal, does this mean that there could be a false negative readout when detecting IgG immune complexes in autoimmune disease sera with Fc gamma receptor I or IIa expressing reporter cells? To this end, do the authors propose that Fc gamma receptor IIIa-expressing reporter cells would be the reporter cell of choice for the detection of pathologic immune complexes?

Reply #11: The reviewer makes a valid point. We feel that we overstated some of the observations made using our reporter system. In addition, some of the previous claims have to be reconsidered in light of the new data (new Fig. 2). We now more clearly state the benefits of the system in experimental and potentially clinical settings throughout the manuscript. We conclude that FCGRIIA(H) and FCGRIIIA would be the most sensitive and suitable reporters for sIC measurement in clinical settings. For FCGRIIIA we provide evidence for a correlation with conventional biomarkers of lupus (Fig. 5).

The authors also state that "As this assay is highly sensitive to any aggregation of IgG, it also represents a tool to control the purity, quality and stability of mAb preparations produced fortherapeutic use in patients." - apologies but I could not find data to support this claim.

Reply #12: The reviewer is right; we did not show data on this aspect. Although we performed experiments in this direction and are convinced that this is a possible application, we now mark this point more clearly as conjecture inspired by the assay's sensitivity towards complexed IgG.

The article is well-written and clear, and key concepts are well explained (notwithstanding some technical facts detailed below). It is my opinion that a non-specialist would both be able to understand the article and find it interesting. However a couple of sentences would benefit for greater explanation - e.g. "functional reproduction of the paradigmatic Heidelberger- Kendall precipitation curve, describing that the molecular size of sICs determined by the antibody:antigen ratio". This concept is described in the abstract also but is not adequately explained.

Reply #13: We changed the wording regarding this aspect throughout the manuscript and made any observations and interpretations clearer (lines 26-28 together with lines 34-37, lines 60-63, line 102, lines 238-242, from line 343).

Similarly this concept is not explained or introduced "Among all type I FcγRs," There are several statements in the introduction that are either incomplete and/or to my mind incorrect. For example - no mention of the binding of complement C1q is given in the following sentence - "and the constant Fc region (Fcγ), which is detected by Fcγ receptors (FcγRs) found on 43 most cells of the immune system". This should be rectified for balance.

Reply #14: We apologize for the omission of information on complement factors. The introduction has been adapted accordingly (lines 48-49). We also added further comments on C1q or C3d assays (lines 102-106, lines 317-324 and from line 361).

"When IgG binds to its antigen ICs are formed, which, depending on the respective antigen, are either cell-bound or soluble (sICs)." - IgG can also bind structural and matrix proteins that are not inherenly cellular or soluble

Reply #15: We agree with the reviewer, which is why we make this visual comparison in Fig. 1C and an experimental comparison in Fig. 1B and Fig. 2D.

This statement is unclear" Firmly fixed cell bound ICs" - what does that mean?

Reply #16: We changed the wording for clarity (line 54-58).

This statement is controversial "FcgR1....is primarily tasked with phagocytosis linked to antigen processing and pathogen clearance" and should be removed. Similarly the statements about the definitive roles of each FcgR are not accurate

or well accepted and should be amended "Activation of FcγRs leads to a variety of cellular effector functions such as antibody-dependent cellular cytotoxicity (ADCC) by natural killer (NK) cells via FcγRIIIA, antibody-dependent cellular phagocytosis (ADCP) by macrophages via FcγRI, cytokine and chemokine secretion by NK cells and macrophages via FcγRIIIA." - many of these statements are isotype, assay and context dependent.

Reply #17: Consulting the literature, we agree that attempting to delineate the roles of individual FcgRs in detail can lead to imprecise or incomplete statements, especially in a relatively concise introduction paragraph. As we now focus more on the methodology, we feel that this detailed information does not add to the quality of the manuscript and chose to make more general statements about the individual FcgRs (from line 64).

As indicated above, some of the themes of the paper have been covered in previously published literature (Stopforth et al. 2018). For this reason, care should maybe be taken with the sentence "...that is able for the first time to selectively detect soluble multimeric immune complexes as discrete ligands of FcγR". However, certainly some novel aspects have been explored in the paper, including the recapitulation of the Heidelberger Kendall precipitation curve, work with primary NK cells, and the detection of mouse IgG immune complexes.

Reply #18: As mentioned in replies #1 to Reviewer 1 and in reply #6, we now point out the technical advances more clearly and explain distinctions to other methods more clearly.

Finally, considering the time required to perform such assays, it would be important to clearly define how the assay would be able to be used in a clinical setting.

Reply #19: We now provide a faster readout by measuring CD69 expression using high-throughput flow cytometry (new Fig. 2). We also now perform IL-2 quantification, which could be used for standardized experimentation (new Fig. 2).

Some referencing is lacking - where did the "monomeric CD20 peptide (aa 141-188)" knowledge come from?

Reply #20: As this is a commercial product, this information is found in the Material and Methods section.

Referee #3 (Remarks for Author):

The authors developed a reporter cell-based system that evaluates FcgR activation by sICs as a tool to quantitate sIC activity. They show that sIC generated in vitro, using synthetic dimeric or trimeric antigens, and present in sera of lupus mice and patients are detected by cell lines expressing the ectodomain of the human FcgR fused to the signaling module of the mouse CD3-zeta chain. They also exploited previously described FcgR reporter cells expressing chimeric mouse FcgRs. The readout of activation is the generation of IL-2 detected in the cell culture. Thus, sIC mediated activation rather than binding to FcgRs per se is being measured.

The experiments are technically well done and shows sensitivity to sIC stoichiometry and size (antibody-antigen ratio). However, the objective for this study is not clear. Is it to identify the FcgRs that do not respond to soluble ICs or to develop a reporter system to detect sICs in clinical samples? Both have caveats as described below.

Reply #21: We thank the reviewer for his/her positive evaluation of the technical soundness of our data and pointing out the unclear nature of our initial manuscript. As mentioned in replies #1 to reviewer 1 and reply #10 to reviewer 2, we substantially revised the manuscript to be consistently focused on the new method and not on the biology of FCGRs, in line with the submission as a methods/resources article.

Using the reporter cell panel expressing individual human and mouse FcgRs, the authors claim that sICs do not activate FcgRI or FcgRIIA. The latter is quite surprising and the authors don't explain how FcgRIIA is not, while FcgRIIB/C is activated by sICs when both FcgRs are highly homologous in their extracellular domain. In this regard, the statement that FcgRIIA is unable to recognize sICs (line 73) is incorrect (i.e. Fossati et al. 2002 and Hubbard et al. 2020 are not accurately quoted).

Reply #22: Based on the reviewer's correct criticism we reinvestigated this aspect extensively. The statements regarding FCGR2 have been extended to include FcgRIIA R and H (lines 185-190), which are now also available in the reporter panel (new Fig. 2). Also, we revisited the initial claim of FCGR2 responsiveness to sICs using improved reporter cells and by adding new reporter cells (FCGR3B and FCGR2A(H)). This showed (new Fig. 2) that our initial claims were exaggerated and therefore we removed this biological interpretation based on our assay platform.

The mouse homolog for human FcgRIIA is mFcgRIII and for human FcgRIII is mFcgRIV. However, despite a lack of activation of FcgRIIA by sICs, mFcgRIV responsiveness tended to be lower and more variable than FcgRIII.

Reply #23: With the new Fig. 2, our data is now more in line with this knowledge. Of course, it remains an open question whether the properties measured with synthetic and clinical sICs for FcgRIIA can be readily transferred to mouse FcgRs (IV vs III). The differences we measured between FcgRIIA (R) and (H) when using molecularly well-defined synthetic sICs suggest that small changes in the ectodomain can have significant effects. Therefore, extrapolations from humans to mice can only be made with extreme caution.

The use of human IgG-antigen pairs that provide dimeric or trimeric antigen are innovative ibut it is not clear that this approach is superior to the NIP-antigen system used by Lux et al 2016. It is also not clear how the current studies advances the field as the principle that the molecular size of sICs dynamically tunes FcgR activation (line 59) was explored by Lux et al. 2016.

Reply #24: The publication by Lux et al was an inspiration to our experiments. We think that our system builds on this initial finding by exploring exactly defined synthetic antibody:antigen pairs that are providing proof-of-concept for immune complexes which would naturally form in humans. In addition, using these highly pure molecules allowed us to perform cross-titrations, which enabled us to measure a more detailed Heidelberger-Kendall-like curve with regard to immune complex bioactivity. To our knowledge, this has not been performed before.

If the objective is to develop the reporter cell-based assay as a clinical tool then the authors need to demonstrate that it outperforms current clinical assays and that it is scalable and high-throughput for clinical labs. In addition, the development of a reporter cell line that expresses multiple FcgRs (as is the case for mammalian leukocytes) would be warranted.

Reply #25: We now provide a faster readout with high-throughput potential by measuring CD69 expression via HTS flow cytometry. As our assay is quite unique measuring sIC bioactivity and no comparable assays for routine research or diagnostic purposes have been described so far, we are not able to compare it to current assays, which measure particular immune complex features as detailed in the revised manuscript (from line 361). Our goal was not to replace conventional assays but to provide a method, which is able to fill a gap in methodology. However, we demonstrate that our assay correlates with conventional lupus disease markers (including commercial C3d ELISA). While the expression of more than one FCGR in a single reporter cell is certainly of future research interest, we see a great advantage in the possibility to measure single FCGR activation, which would not be possible with most immune cells. We also think that FCGR co-expression would require native signaling, as our reporter system would not be able to mimic the important receptor interplay seen for example between FCGR2A and FCGR3B on neutrophils.

4th Oct 2021

Dear Dr. Kolb,

Thank you for the submission of your manuscript to EMBO Molecular Medicine. We have now received feedback from the two reviewers who agreed to re-evaluate your manuscript. As you will see from the reports below referee #2 supports publication of your work, while referee #1 acknowledges your efforts in addressing previous comments but also raises several concerns that should be addressed in a revision of the current manuscript.

EMBO Molecular Medicine encourages a single round of revision only and therefore, acceptance or rejection of the manuscript will depend on the completeness of your responses included in the next, final version of the manuscript. For this reason, and to save you from any frustrations in the end, I would strongly advise against returning an incomplete revision. Please format your manuscript following EMBO Molecular Medicine "Author Guidelines" for "Manuscript Preparation" and "Submission of Revisions". https://www.embopress.org/page/journal/17574684/authorguide#manuscriptpreparation
https://www.embopress.org/page/journal/17574684/authorguide#submissionofrevisions

We would welcome the submission of a revised version within three months for further consideration. Please let us know if you require longer to complete the revision.

I look forward to receiving your revised manuscript.

Yours sincerely,

Zeljko Durdevic

Zeljko Durdevic
Editor
EMBO Molecular Medicine

***** Reviewer's comments *****

Referee #1 (Comments on Novelty/Model System for Author):

A novel system to evaluate the immune complex-mediated activation via human Fcgamma receptors

Referee #1 (Remarks for Author):

Authors have edited the manuscript by considering the previous comments.Couple of additional comments following their revision.

1. Authors should provide a comparison of severity score of SLE patients based on the clinical parameters that are followed and their assay readout

2. Should perform an assay where authors could use intravenous immunoglobulin (IVIG) to block the activation of Fcgamma receptors expressing cells by immune complexes.

3. Control experiments by using F(ab')2 fragments of IgG to form an immune complex and analyzing the activation status of cell line.

Referee #2 (Comments on Novelty/Model System for Author):

The authors have answered all/ most of the comments satisfactorily, toned down the claims of novelty, improved the legends, showed quantification of IL-2 for the ELISAs, added the CD69 readout and included a CD32A vs H comparison etc. They no longer seem to make conclusions about individual FcgRs and seem to write it more as a methods paper which accurately reflects the findings

Referee #2 (Remarks for Author):

Thanks for your efforts in revising the manuscript; it was clearly a lot of work. You have answered all/ most of the comments satisfactorily, toned down the claims of novelty, improved the legends, showed quantification of IL-2 for the ELISAs, added the CD69 readout and included a CD32A vs H comparison etc. I hope people now take on your method and evaluate it fully

Point by point reply to reviewers' comments:

Referee #1 (Remarks for Author):

Authors have edited the manuscript by considering the previous comments. Couple of additional comments following their revision.

1. Authors should provide a comparison of severity score of SLE patients based on the clinical parameters that are followed and their assay readout

Reply#1: We agree that this information would strengthen the validity of our study and complement the current correlations with individual biomarkers of SLE. Therefore, we compared patient SLEDAI values with our assay measurements (new Fig 5C). This showed a significant correlation with FcgRIIIA (p=0.0109), but not FcgRIIB (p=0.4979), in line with our previous observations now found in Fig. 5D.

2. Should perform an assay where authors could use intravenous immunoglobulin (IVIG) to block the activation of Fcgamma receptors expressing cells by immune complexes.

Reply#2: The reviewer makes an excellent point. However, according to the literature, IVIg does not necessarily block FcgR activation by sICs (reviewed in doi:10.1038/nri3401). In addition, FcgRs should have a higher affinity towards sICs compared to non-complexed IgG (doi: 10.1182/blood-2008-09-179754). Accordingly, when we performed such an experiment, sIC activation of our reporter cells was not compromised by the addition of IVIg (new Fig. EV1C). This in turn leads us to the conclusion that our assay is well-suited for the detection of sICs even in the presence of large amounts of non-complexed IgG, which we would find for example in patient serum. This also explains how we can still detect sICs with high sensitivity in patient serum (Fig. 5).

3. Control experiments by using F(ab')2 fragments of IgG to form an immune complex and analyzing the activation status of cell line.

Reply#3: We now provide such a control experiment in Fig.EV1B. This shows that TNF/Ifx[F(ab)2] sICs cannot activate FcgR3a, while TNF/Ifx sICs can. As expected, this now directly shows that Fc-gamma has to be present in the sICs in order to activate our reporter cells.

Referee #2 (Remarks for Author):

Thanks for your efforts in revising the manuscript; it was clearly a lot of work.

You have answered all/ most of the comments satisfactorily, toned down the claims of novelty, improved the legends, showed quantification of IL-2 for the ELISAs, added the CD69 readout and included a CD32A vs H comparison etc. I hope people now take on your method and evaluate it fully

Reply#4: We thank the reviewer again for the entirely constructive criticism, which was key to the major improvement of the manuscript.

5th Nov 2021

Dear Dr. Kolb,

Thank you for the submission of your revised manuscript to EMBO Molecular Medicine. I am pleased to inform you that we will be able to accept your manuscript pending the following final amendments:

1) In the main manuscript file, please do the following:
- Make sure that all special characters display well.
- In M&M, statistical paragraph should reflect all information that you have filled in the Authors Checklist, especially regarding randomization, blinding, replication etc.
- In M&M, include a statement that informed consent was obtained from all human subjects and that in addition to the WMA Declaration of Helsinki the experiments also conformed to the principles set out in the Department of Health and Human Services Belmont Report.
2) Synopsis: Please check your synopsis text and image before submission with your revised manuscript. Please be aware that in the proof stage minor corrections only are allowed (e.g., typos).
3) For more information: There is space at the end of each article to list relevant web links for further consultation by our readers. Could you identify some relevant ones and provide such information as well? Some examples are patient associations, relevant databases, OMIM/proteins/genes links, author's websites, etc...
4) As part of the EMBO Publications transparent editorial process initiative (see our Editorial at http://embomolmed.embopress.org/content/2/9/329), EMBO Molecular Medicine will publish online a Review Process File (RPF) to accompany accepted manuscripts. This file will be published in conjunction with your paper and will include the anonymous referee reports, your point-by-point response and all pertinent correspondence relating to the manuscript. Let us know whether you agree with the publication of the RPF and as here, if you want to remove or not any figures from it prior to publication. Please note that the Authors checklist will be published at the end of the RPF.
5) Please provide a point-by-point letter INCLUDING my comments as well as the reviewer's reports and your detailed responses (as Word file).

I look forward to reading a new revised version of your manuscript as soon as possible.

Yours sincerely,

Zeljko Durdevic

Zeljko Durdevic
Editor
EMBO Molecular Medicine

e t s pe e t e e este e t es

We are pleased to inform you that your manuscript is accepted for publication and is now being sent to our publisher to be included in the next available issue of EMBO Molecular Medicine.

EMBO PRESS

YOU MUST COMPLETE ALL CELLS WITH A PINK BACKGROUND

PLEASE NOTE THAT THIS CHECKLIST WILL BE PUBLISHED ALONGSIDE YOUR PAPER

Corresponding Author Name: Philipp Kolb	
Journal Submitted to: EMBO Molecular Medicine	
Manuscript Number: EMM-2021-14182-V4-Q	

Reporting Checklist For Life Sciences Articles (Rev. June 2017)

This checklist is used to ensure good reporting standards and to improve the reproducibility of published results. These guidelines are consistent with the Principles and Guidelines for Reporting Preclinical Research issued by the NIH in 2014. Please follow the journal's authorship guidelines in preparing your manuscript.

A- Figures

1. Data

The data shown in figures should satisfy the following conditions:

- the data were obtained and processed according to the field's best practice and are presented to reflect the results of the experiments in an accurate and unbiased manner.
- figure panels include only data points, measurements or observations that can be compared to each other in a scientifically meaningful way.
- graphs include clearly labeled error bars for independent experiments and sample sizes. Unless justified, error bars should not be shown for technical replicates.
- if n< 5, the individual data points from each experiment should be plotted and any statistical test employed should be justified
- Source Data should be included to report the data underlying graphs. Please follow the guidelines set out in the author ship guidelines on Data Presentation.

2. Captions

Each figure caption should contain the following information, for each panel where they are relevant:

- a specification of the experimental system investigated (eg cell line, species name).
- the assay(s) and method(s) used to carry out the reported observations and measurements
- an explicit mention of the biological and chemical entity(ies) that are being measured.
- an explicit mention of the biological and chemical entity(ies) that are altered/varied/perturbed in a controlled manner.
- the exact sample size (n) for each experimental group/condition, given as a number, not a range;
- a description of the sample collection allowing the reader to understand whether the samples represent technical or biological replicates (including how many animals, litters, cultures, etc.).
- a statement of how many times the experiment shown was independently replicated in the laboratory.
- definitions of statistical methods and measures:
  - common tests, such as t-test (please specify whether paired vs. unpaired), simple χ2 tests, Wilcoxon and Mann-Whitney tests, can be unambiguously identified by name only, but more complex techniques should be described in the methods section;
  - are tests one-sided or two-sided?
  - are there adjustments for multiple comparisons?
  - exact statistical test results, e.g., P values = x but not P values < x;
  - definition of 'center values' as median or average;
  - definition of error bars as s.d. or s.e.m.

Any descriptions too long for the figure legend should be included in the methods section and/or with the source data.

In the pink boxes below, please ensure that the answers to the following questions are reported in the manuscript itself. Every question should be answered. If the question is not relevant to your research, please write NA (non applicable). We encourage you to include a specific subsection in the methods section for statistics, reagents, animal models and human subjects.

USEFUL LINKS FOR COMPLETING THIS FORM

http://www.antibodypedia.com
http://1degreebio.org
http://www.equator-network.org/reporting-guidelines/improving-bioscience-research-repor
http://grants.nih.gov/grants/olaw/olaw.htm
http://www.mrc.ac.uk/Ourresearch/Ethicsresearchguidance/Useofanimals/index.htm
http://ClinicalTrials.gov
http://www.consort-statement.org
http://www.consort-statement.org/checklists/view/32-consort/66-title
http://www.equator-network.org/reporting-guidelines/reporting-recommendations-for-tumo
http://datadryad.org
http://figshare.com
http://www.ncbi.nlm.nih.gov/gap
http://www.ebi.ac.uk/ega
http://biomodels.net/
http://biomodels.net/miriam/
http://jjj.biochem.sun.ac.za
https://osp.od.nih.gov/biosafety-biosecurity-and-emerging-biotechnology/
http://www.selectagents.gov/

B- Statistics and general methods

Please fill out these boxes (Do not worry if you cannot see all your text once you press return)

1.a. How was the sample size chosen to ensure adequate power to detect a pre-specified effect size?	Primary NK cells and Neutrophils were isolated from three or four healthy donors to enable statistical analysis and ensure reproducibility across donors. 25 SLE patients were compared to four healthy control sera. This was chosen as an adequate sample size to allow for statistical analyses and to detect convincing trends in our data.
1.b. For animal studies, include a statement about sample size estimate even if no statistical methods were used.	three individual mice from each genetic background were chosen as an adequate sample size to show clear trends in our proof-of-concept experiments.
2. Describe inclusion/exclusion criteria if samples or animals were excluded from the analysis. Were the criteria pre-established?	SLE patients with sub-detection levels anti-dsDNA or C3d were excluded from Figure 5 D as described in the figure legend.
3. Were any steps taken to minimize the effects of subjective bias when allocating animals/samples to treatment (e.g. randomization procedure)? If yes, please describe.	samples or animals were not chosen on any bias. Samples were tested as they were available.
For animal studies, include a statement about randomization even if no randomization was used.	no randomization was used
4.a. Were any steps taken to minimize the effects of subjective bias during group allocation or/and when assessing results (e.g. blinding of the investigator)? If yes please describe.	n.a.
4.b. For animal studies, include a statement about blinding even if no blinding was done	no blinding was done
5. For every figure, are statistical tests justified as appropriate?	yes
Do the data meet the assumptions of the tests (e.g., normal distribution)? Describe any methods used to assess it.	Data was analyzed using Prism software - Multiple comparison ANOVA. Correction as indicated
Is there an estimate of variation within each group of data?	Error bars or 95%Cb are shown in the respective figures

Is the variance similar between the groups that are being statistically compared?	Multiple comparison by ANOVA.

C- Reagents

6. To show that antibodies were profiled for use in the system under study (assay and species), provide a citation, catalog number and/or clone number, supplementary information or reference to an antibody validation profile. e.g., Antibodypedia (see link list at top right), 1DegreeBio (see link list at top right).	Antibodies are detailed in the manuscript material and methods section. FcγR-specific mAbs were obtained from Stem Cell technologies (CD16: clone 3G8; CD32: IV.3). Mouse anti-hTNFα (IgG2b, R&D Systems, 983003) was used to generate sICs reactive with mouse FcγRs. infliximab (Ifx), bevacizumab (Bvz), mepolizumab (Mpz) and rituximab (Rtx) were obtained from the University Hospital Pharmacy Freiburg.
7. Identify the source of cell lines and report if they were recently authenticated (e.g., by STR profiling) and tested for mycoplasma contamination.	cells were routinely tested for mycoplasma using an in-house PCR-based method and used if negative. Sources of cell lines are detailed in the material and methods section of the manuscript. Cells were not authenticated.

* for all hyperlinks, please see the table at the top right of the document

D- Animal Models

8. Report species, strain, gender, age of animals and genetic modification status where applicable. Please detail housing and husbandry conditions and the source of animals.	mus musculus. Lupus-prone (NZBxNZW)F1 mice (NZB/WF1) were generated by crossing NZB/BlNJ mice with NZW/LacJ mice, purchased from The Jackson Laboratory. KRNtg mice were obtained from F. Nimmerjahn (Universität Erlangen-Nürnberg) with the permission of D. Mathis and C. Benoist (Harvard Medical School, Boston, MA), C57BL/6 mice (BL/6) and NOD/ShiLtJArc (NOD/Lt) mice were obtained from the Charles River Laboratories. K/BxN (KRNtgxNOD)F1 mice (K/BxN) were obtained by crossing KRNtg mice and NOD/Lt mice. All mice were housed in a 12-h light/dark cycle, with food and water ad libitum.
9. For experiments involving live vertebrates, include a statement of compliance with ethical regulations and identify the committee(s) approving the experiments.	Animal experiments were approved by the local governmental commission for animal protection of Freiburg (Regierungspräsidium Freiburg, approval no. G16/59 and G19/21)
10. We recommend consulting the ARRIVE guidelines (see link list at top right) (PLoS Biol. 8(6), e1000412, 2010) to ensure that other relevant aspects of animal studies are adequately reported. See author guidelines, under 'Reporting Guidelines'. See also: NIH (see link list at top right) and MRC (see link list at top right) recommendations. Please confirm compliance.	confirmed

E- Human Subjects

11. Identify the committee(s) approving the study protocol.	Sera from patients with SLE were obtained from the Immunologic, Rheumatologic Biobank (IR-B) of the Department of Rheumatology and Clinical Immunology. Biobanking and the project were approved by the local ethical committee of the University of Freiburg (votes 507/16 and 624/14).
12. Include a statement confirming that informed consent was obtained from all subjects and that the experiments conformed to the principles set out in the WMA Declaration of Helsinki and the Department of Health and Human Services Belmont Report.	All patients who provided blood to the biobank had provided written informed consent. The study was designed in accordance with the guidelines of the Declaration of Helsinki (revised 2013)
13. For publication of patient photos, include a statement confirming that consent to publish was obtained.	n.a.
14. Report any restrictions on the availability (and/or on the use) of human data or samples.	Samples were taken from a biobank and are therefore limited in volume. We accordingly did not test all reporter cells with SLE patient serum.
15. Report the clinical trial registration number (at ClinicalTrials.gov or equivalent), where applicable.	n.a.
16. For phase II and III randomized controlled trials, please refer to the CONSORT flow diagram (see link list at top right) and submit the CONSORT checklist (see link list at top right) with your submission. See author guidelines, under 'Reporting Guidelines'. Please confirm you have submitted this list.	n.a.
17. For tumor marker prognostic studies, we recommend that you follow the REMARK reporting guidelines (see link list at top right). See author guidelines, under 'Reporting Guidelines'. Please confirm you have followed these guidelines.	n.a.

F- Data Accessibility

18: Provide a "Data Availability" section at the end of the Materials & Methods, listing the accession codes for data generated in this study and deposited in a public database (e.g. RNA-Seq data: Gene Expression Omnibus GSE39462, Proteomics data: PRIDE PXD000208 etc.) Please refer to our author guidelines for 'Data Deposition'.<br><br>Data deposition in a public repository is mandatory for:<br>a. Protein, DNA and RNA sequences<br>b. Macromolecular structures<br>c. Crystallographic data for small molecules<br>d. Functional genomics data<br>e. Proteomics and molecular interactions	n.a.
19. Deposition is strongly recommended for any datasets that are central and integral to the study; please consider the journal's data policy. If no structured public repository exists for a given data type, we encourage the provision of datasets in the manuscript as a Supplementary Document (see author guidelines under 'Expanded View' or in unstructured repositories such as Dryad (see link list at top right) or Figshare (see link list at top right).	n.a.
20. Access to human clinical and genomic datasets should be provided with as few restrictions as possible while respecting ethical obligations to the patients and relevant medical and legal issues. If practically possible and compatible with the individual consent agreement used in the study, such data should be deposited in one of the major public access-controlled repositories such as dbGAP (see link list at top right) or EGA (see link list at top right).	n.a.
21. Computational models that are central and integral to a study should be shared without restrictions and provided in a machine-readable form. The relevant accession numbers or links should be provided. When possible, standardized format (SBML, CellML) should be used instead of scripts (e.g. MATLAB). Authors are strongly encouraged to follow the MIRIAM guidelines (see link list at top right) and deposit their model in a public database such as Biomodels (see link list at top right) or JWS Online (see link list at top right). If computer source code is provided with the paper, it should be deposited in a public repository or included in supplementary information.	n.a.

G- Dual use research of concern

22. Could your study fall under dual use research restrictions? Please check biosecurity documents (see link list at top right) and list of select agents and toxins (APHIS/CDC) (see link list at top right). According to our biosecurity guidelines, provide a statement only if it could.	n.a.
